# The Field of Science

**Published:** 1894-06-16

**Authors:** 


### The Field of Science.

The Council of the University of Liverpool has received the generous offer from Mr. George Holt of £10,000 towards the object of endowing a chair of Pathology in the Medical School. The only endowed chair hitherto possessed by the school is that of Physiology, which also was a gift from the same donor.

We learn that the Government of India is continuing to make systematic inquiries into the question of how far strychnine is efficacious in the treatment of snake bites. A list of cases thus treated during the last twelve months has been forwarded to the Punjab Government for further investigation.

The New Jersey State Hospital for the Insane is doing some good work in the Pathological Laboratory in connection with this institution. No less than thirty-eight autopsies were made by the pathologist in residence. Among this number a case of choreic insanity was discovered. "Granular degeneration with vacuolation" were shown to be features of some prominence in this microscopical examination of the brain cells in this case. This vacuolation, it was pointed out in the report of the work done in the laboratory, was a condition frequently observed in the study of the effect of normal fatigue on brain cells of insects and animals of the lower order.

M. Pasteur expressed himself the other night at the Paris Council of Hygiene and Salubrity in a manner it would be well if the authorities would act upon. The question of the disposal of specified sewage was under discussion. The learned scientist commented thus: "It is proposed," he said, "not to conduct to the sea the pathogenic germs of the numerous contagious diseases which decimate all our population, but to accumulate them each year more and more on the fields situate at the gate of the great town, and these fields will be cultivated. It would be better if the fields remained uncultivated, for then you would not incur the risks of bringing back the germs to Paris." Surely but little common sense is required to see the significance of M. Pasteur's comment, and what the results will be if this practice be continued. Quite recently a contemporary quoted cases of cholera occurring at Havre, which have been actually traced to the eating of vegetables so treated.

The gold medal of the Linnæan Society has been awarded to Professor Haeckel, of Jena. The award was consequent on the Professor's many contributions and additions to zoological science.

The Danish Government have decided on supplying the necessary funds for an exhaustive investigation on the study of leprosy in Iceland. The commission includes two French physicians, Dr. Eichmuller and Dr. Neisser, both of Paris. It is reported that 50 cases of the disease have been registered in Iceland, and we shall await with much interest further information from the commission. It is predicted fresh light will very possibly be hereby thrown upon the etiology and pathology of leprosy.

One would like to ask what are the French water-drinking public to do, now that their faith in the safety of their "siphon" has been so rudely shaken. There has always been much to say in favour of this siphon, since the Paris "drinking" water itself was rather too closely allied to typhoid and other diseases. And now the Académie de Médicine has contaminated the great safety valve of the blue ribbon portion of the public. No, we mean has proved their siphon contaminated! What is to take its place? For to know a thing is harmful, and to do it, is proverbially ever more conducive of bad results than if the deed is accomplished in an ignorant state of mind.

Poor siphon drinkers who cheated themselves into a belief in the purity of their beverage, now learn on the authority of a most august body of investigators that a bad case is proven against the "purity" of these "natural" mineral waters. Apollinaris Water is exceptional, however. This appears to have stood the severest test brought to bear on it, and to have come out in a quite triumphal manner, and there were here found no traces of liquifying colonies of bacilli whatsoever. This is more than the other waters can say for themselves? Take the "natural waters" of Couzan for instance, one sample of which contained as many as 200 liquifying colonies! and Vichy contained them in even more excessive a quantity. It has been pointed out that the contamination is due in many instances to the actual bottling of the water. The Académie de Médicine suggest, alas! no substitute for the siphon, of the faith in which it has completely robbed the Parisian.

